# The Road to Survival for Haemodynamically Unstable Patients With Open Pelvic Fractures

**DOI:** 10.3389/fsurg.2020.00058

**Published:** 2020-09-02

**Authors:** Rachel J. Watkins, Jeremy M. Hsu

**Affiliations:** ^1^Trauma Service, Westmead Hospital, Westmead, NSW, Australia; ^2^Discipline of Surgery, University of Sydney, Sydney, NSW, Australia

**Keywords:** open pelvic fracture, perineal injury, pelvic ring fracture, haemodynamic instability, rectal injury, urogenital injuries, extraperitoneal packing, trauma

## Abstract

Management of haemodynamically unstable pelvic ring injuries has been simplified into treatment algorithms to streamline care and emergent decision making in order to improve patient outcomes whilst decreasing mortality and morbidity. Pelvic ring injuries are most commonly a result of high-velocity and energy forces that exert trauma to the pelvic bones causing not only damage to the bone but the surrounding soft-tissue, organs, and other structures and are usually accompanied by injuries to other parts of the body resulting in a polytraumatised patient. Open pelvic fractures are a rare subset of pelvic ring fractures that are on the more severe end of the pelvic fracture continuum and usually produce uncontrolled haemorrhage from fractured bone, retroperitoneal haematomas, intraabdominal bleeding from bowel injury, soft tissue injuries to the anus, perineum, and genitals, fractures of the pelvic bones, causing bleeding from cancellous bone, venous, and arterial injuries combined with bleeding from concomitant injuries. This is a very complex and challenging clinical situation and timely and appropriate decisions and action are paramount for a positive outcome. Consequently, open pelvic fractures have an extremely high rate of mortality and morbidity and outcomes remain poor, despite evidence-based improvements in treatment, knowledge, and identification of haemorrhage; in the pre-hospital, critical care, and operative settings. In the future utilisation of haemostatic drugs, dressings, devices, and procedures may aid in the time to haemorrhage control.

## Introduction

Open pelvic fractures are a rare subset of pelvic ring fractures, comprising 2–4% of all pelvic ring fractures (1, 2), that are the most severe, debilitating, and life-threatening of all the pelvic ring injuries. There is considerably high but also variable mortality reported with open pelvic fractures, between 4 and 41.8% (3–5). High-energy trauma is required to produce open pelvic fractures. These patients are more likely to be haemodynamically unstable, as they are often polytraumatised, with multiple concomitant injuries that are challenging to collectively manage.

Open pelvic fractures, defined as a fracture of the pelvic bones that communicates with the external environment, through a wound in the adjacent soft tissue; skin, mucosa, vagina, or rectum (1, 3). Bladder and urethral injuries are very common in patients with open pelvic fractures (6) however, associated injuries to the; thoracic spine, abdomen, head, and extremities are also likely (1, 3, 6, 7). Age >65 (1, 7–10), fracture instability (1, 8, 10), revised trauma score (RTS) <8 (7, 8, 11, 12), hypotension and shock on arrival (7, 9, 12), large wounds and contamination (1, 10), Glasgow Coma Scale (GCS) <8 (7, 8), rectal injury (1, 10, 11, 13), and the amount of blood transfused in the first 24 h (1, 7, 8) were identified in the literature as factors that contribute to mortality in these patients.

Open pelvic fractures most frequently affect; previously healthy young males, causing chronic functional impairment and reduced quality of life (QOL) (14). The average Injury Severity Score (ISS) reportedly ranges from 21 to 46, demonstrating the presence of multiple severe injuries (1, 3, 4, 7, 8, 13). Collisions involving; motor bikes, motor vehicles, and pedestrian, are responsible for most open pelvic fractures, with motor bike collisions being the most common mechanism (3, 4, 6, 7, 10, 11, 14, 15). Significantly more males were reported to have open pelvic fractures (3, 4, 6–8, 10, 13, 15), with hospital length of stay (LOS) averages of between 44.1 and 60 days (4, 8).

Mortality occurs early in the acute phase due to exsanguination from uncontrolled haemorrhage (6, 9, 10, 16–20) or later from sepsis causing multi-organ failure (MOF) due to pelvic infection from contaminated perineal and rectal wounds (4, 6, 9, 11, 13, 18). The Western Trauma Association (WTA) algorithm for the management of pelvic fractures with haemodynamic instability was published in 2008 and has become the mainstay for management of these patients. This modern evidence-based approach encompasses a multidisciplinary team approach involving input from trauma surgeons, orthopaedic surgeons interventional radiologists, plastic surgeons, urological surgeons, wound-care nurses, and rehabilitation physicians to optimise outcome through the co-ordination and prioritisation of care, from evaluation, to stabilisation and treatment of the polytraumatised patient (21, 22).

## Management

The management of a haemodynamically unstable patient with open pelvic fractures focuses on identifying the source of bleeding, followed by haemorrhage control. This is subsequently followed by definitive management and repair. A systematic and controlled method of assessing, diagnosing, and prioritising treatment should be adhered to prevent missed injuries and guide the multidisciplinary team. Grotz et al. (1) proposed that management of open pelvic fractures should be divided into five phases*: (1) Haemorrhagic phase; (2) Diagnostic phase; (3) Early Treatment phase; (4) Definitive Treatment phase; (5) Recovery phase*. For the purpose of reviewing the management of haemodynamically unstable patients we will examine the first three phases; haemorrhagic, diagnostic, and early treatment phases. These phases are not exclusive as they often occur simultaneously in the hospital setting.

### Haemorrhagic Phase

In the last decade advancement in systems and technology to identify and treat life-threatening pelvic bleeding and associated injuries has improved the survivability of open pelvic fractures (23). Reducing time to treatment through hospital bypass and rapid transport to level 1 trauma hospitals, and improved pre-hospital protocols, enables the haemodynamically unstable patient with open pelvic fracture to reach the hospital alive (16).

Prehospital treatment has become more sophisticated and methodical, addressing the haemorrhagic phase and preventing early mortality due to exsanguination prior to hospital admission. Time is critical and rapid identification and treatment of haemorrhagic shock with the activation of medical retrieval teams and aeromedical transport to a level 1 trauma centre that is equipped to treat these extensive and resource intensive injuries is essential (16, 24). The availability of pre-hospital blood products and tranexamic acid (TXA), and the adoption of principles such as permissive hypotension (to avoid clot disruption prior to bleeding control), and avoidance of haemodilution by minimising crystalloid infusion [to prevent trauma induced coagulopathy (TIC) through haemodilution of clotting factors], has been adopted as the standard treatment of these patients enabling them to tolerate being transported to a level 1 trauma centre (25). Other treatments that have been widely implemented and have shown benefit include; preventing the lethal triad of coagulopathy and haemorrhage by reducing the patients exposure and preventing hypothermia (26). Temporary pelvic stabilisation using a pelvic binder to reduce and stabilise the fractured pelvic bones, preventing movement, and bleeding from the fracture sites, can aid in haemorrhage control. Controversy surrounds the use of pelvic binding on lateral compression type injuries as they can become more haemodynamically unstable with the application of a binder due to the pelvis effectively collapsing inwards (27) and open pelvic fractures may also pose an impediment to binder application depending on the degree of soft tissue disruption. There are also a variety of haemostatic agents that can be used pre-hospital and during surgery to arrest bleeding including; systemic and topical haemostatic agents (24, 28). Systemic haemostatic agents including; intravenous infusion of blood products, coagulation factors, and TXA, have become widely accepted, along with the guidance of a Haematologist to manage blood product administration in the haemorrhaging patient. Topical haemostatics are used more in combat situations, these are more useful prehospital and are not as commonly employed as part of the surgical management of these patients. They include; dressings impregnated with haemostatic agents (Chitosan, Zeolite, Combat gauze, Quickclot, Kaolin impregnated gauze) and injectable and self-expanding sponges (Xstat-multiple radiopaque expanding mini-sponges) (24, 28).

### Diagnostic Phase

Advances in emergency critical care, a multidisciplinary team response and the adoption of new principles and technology have improved the in-hospital mortality rates in patients with open pelvic fractures moving them from the life-threating haemorrhagic phase to the diagnostic and early treatment phases. Massive transfusion protocols (MTP) and changes to blood product ratios (closer to that of whole blood) encourage blood product administration in the haemodynamically unstable patient allowing quick and readily available blood product administration (29, 30). The use of new technology such as viscoelastic coagulation testing Thromboelastography (TEG) and thromboelastometry (ROTEM) to guide blood product administration and the adoption of TEG/ROTEM guided MTP algorithms to identify TIC are becoming more popular in major trauma centres (31, 32).

Potential sources of bleeding must be identified, as many of these patients are polytraumatized and therefore may have multiple sites of haemorrhage. Assessment of the source of bleeding is essential in haemodynamically unstable patients to guide treatment in our institution. Ruling out abdominal bleeding quickly with an extended focused assessment with sonography for trauma (e-FAST) or Diagnostic Peritoneal Aspirate (DPA) in haemodynamically unstable patients is common practice guiding rapid decision-making regarding patient destination from the Emergency Department and the need for a trauma laparotomy (33, 34). A chest x-ray or e-FAST should be performed to assess for intrathoracic bleeding.

**Figure 1:**
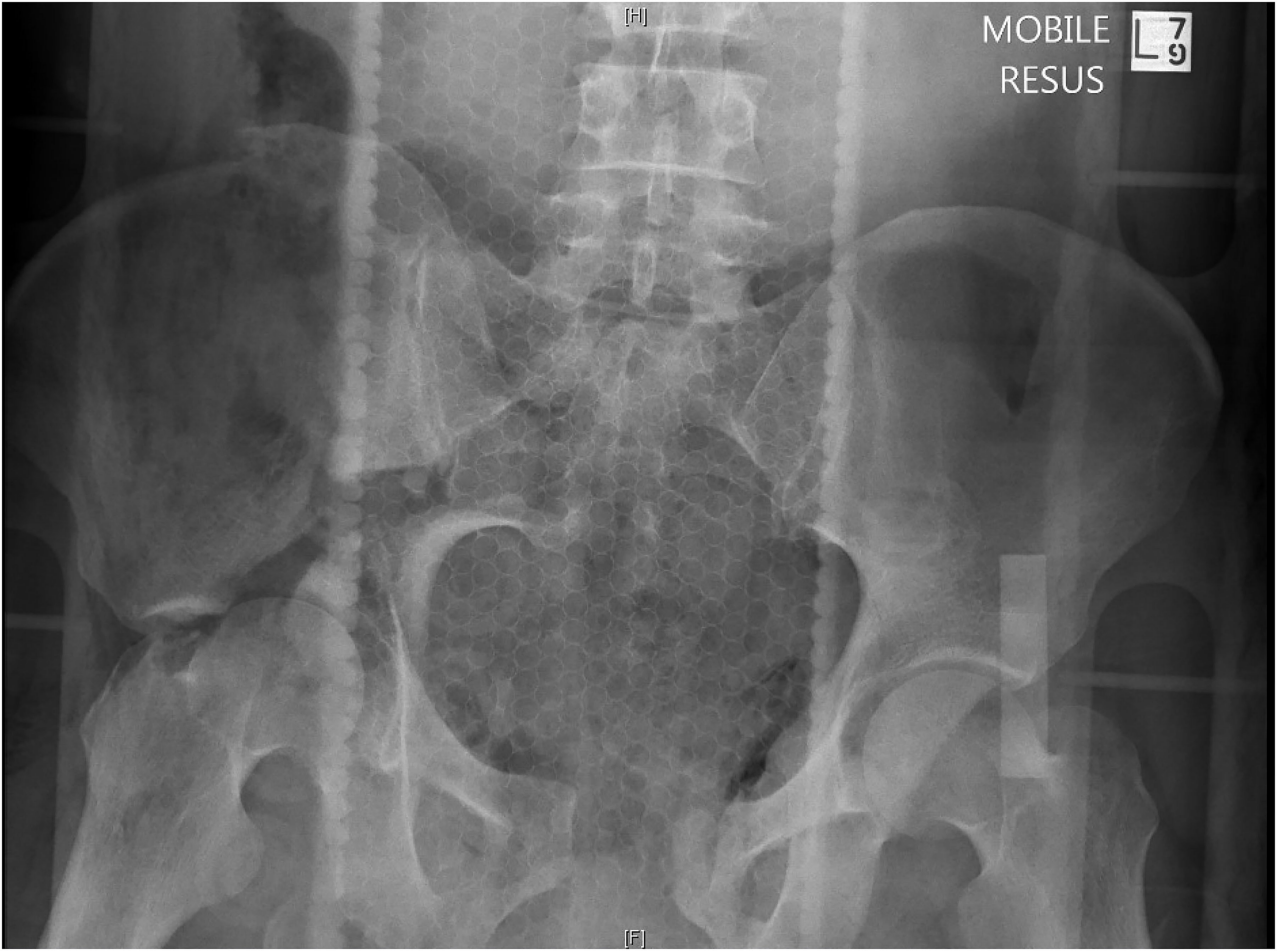
Pelvic x-ray in Emergency Department- 38-year-old male, motor bike vs. car.

A pelvic x-ray should be performed in the emergency room (as an adjunct to the primary survey) to identify the type and severity of the pelvic fractures, which will help guide critical decision making in the haemodynamically unstable patient (Figure 1). Pelvic fractures are classified according to the Tile or Young and Burgess classification systems. The Tile classification system is based on the stability of the pelvis; A-stable, B-rotationally unstable, or C- rotationally and vertically unstable. The Young and Burgess classifies pelvic fractures according to the MOI; lateral compression (LC), anteroposterior compression (AP), or vertical shear (VS). Both have similar predictive value for significant bleeding and mortality (35).

**Figure 2:**
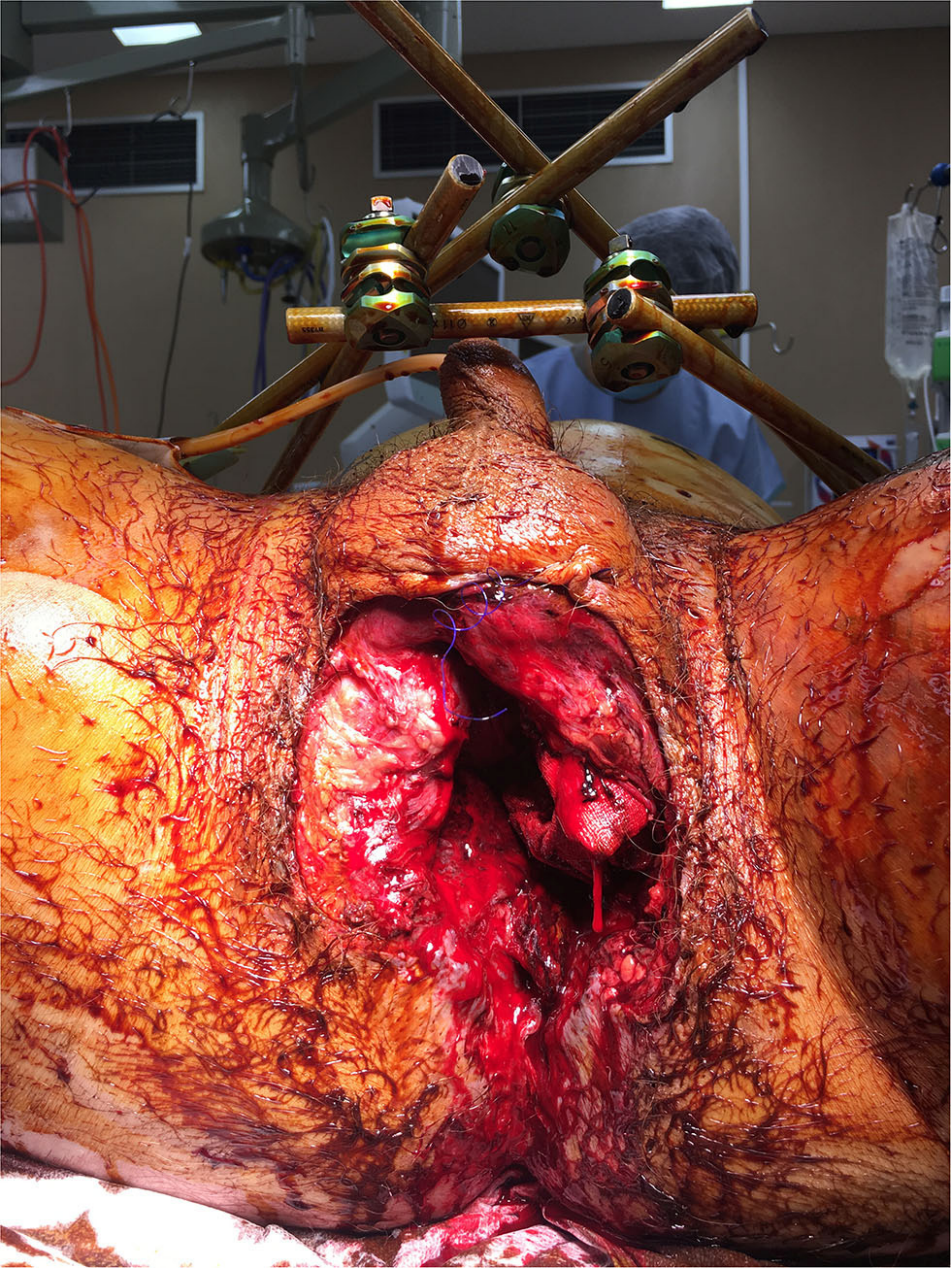
Intraoperative photo showing massive pelvic floor and perianal disruption from open pelvic fractures.

A thorough physical examination should be conducted including; inspection of the patient's genitals, perineum, and buttocks (1). In the haemodynamically unstable patient this will be performed in the operating room. A digital rectal exam (DRE), and a vaginal exam (PV) in females, should also be performed to identify rectal, bowel, and other communicating pelvic wounds (1). DRE to check for a high-riding prostate in males is thought to be obsolete as this assessment has been found to be unreliable for evaluation of urethral disruption in males (36). Blood at the external urethral meatus, gross haematuria, or in females- vaginal lacerations or bleeding, increases suspicion for bladder or urethral injuries and a suprapubic catheter (SPC) can be inserted in the operating theatre, to drain the bladder (1, 37). The associated soft tissue injuries (STI) are classified according to the Faringer or the Jones-Powell classification systems (Table 1). The zones were considered a guide for faecal diversion surgery and the wounds were also described in terms of their depth—superficial, deep, and avulsions or degloving (15). Grading of open pelvic fractures according to the Jones-Powell classification combined pelvic ring stability and the presence/absence of perineal and rectal wounds. Cannada et al. (13) reported an increased mortality of 38% for class 3 open pelvic fractures compared to an overall mortality of 23% (13). Thus, classification of STI is important for understanding the severity and extent of injury, the need for faecal diversion and potential outcome and mortality (1, 13, 15). Figure 2 demonstrates the perineal injury associated with a major open pelvic fracture.

**Table 1:** Classification systems for open pelvic fractures.

**Zone**	**Anatomical region**
I	Anteriorly from the pubis, perineum-bordered by the inguinal creases, to the posterior sacrum including the medial aspect of the buttocks bilaterally.
II	Medial thighs, including the groin creases, bounded laterally on the anterior thigh by a line drawn between the anterior superior iliac spine to the medial patella inferiorly and posteriorly by the mid-thigh.
III	Posterolateral buttock, iliac crest buttock inferior to the iliac crest.
**Class**	**Description**
1	Stable pelvic ring
2	Pelvic ring unstable- no rectal or perineal wound
3	Pelvic ring unstable- rectal and/or perineal wound

*Faringer (15) and Jones-Powell (19)*.

Computerised tomography (CT) is also useful to determine bone, organ, soft tissue injury, and active bleeding shown as “blush” using CT angiogram with contrast, but should be avoided in a patient who is in extremis and should be performed after the patient has been stabilised (1, 4, 7, 16, 21, 22, 32, 38).

### Early Treatment Phase

The use of resuscitative endovascular balloon occlusion of the aorta (REBOA) is also a controversial temporising measure to manage haemorrhagic shock prior to gaining definitive haemostasis which requires access to the operating theatre and interventional radiology (which can sometimes take time to activate) (32, 39). Temporary fracture stabilisation, angioembolisation, and extraperitoneal packing (EPP) are used to manage the haemorrhagic phase for patients with pelvic ring fractures (22, 32–34, 40–42), however the technique and the order in which they are applied is determined by the surgeon, resources, and institution. In addition to these management options soft tissue injuries to the perineum, genitals, bladder, and bowel need to be considered and managed in patients with open pelvic fractures, complicating the treatment, and decision making in this group of patients. In open pelvic fractures fracture stabilisation and angioembolisation remain relevant, however open pelvic ring fractures are not always amenable to EPP as the retroperitoneal space is open and hence a tamponade effect would be difficult to obtain due to extensive soft tissue injury and pelvic floor deformity. Packing points of haemorrhage externally may be more successful in stemming haemorrhage. Therefore, it may be more useful in the case of extensive open pelvic fractures, to pack from the outside-in for these wounds rather than in the extraperitoneal space, to provide the required pressure and desired tamponade effect to stop haemorrhage (Figure 2). Packing may also be modified by inserting pads into the prevesical and presacral spaces (1, 42, 43).

Success in the diagnostic and early treatment phases has been influenced by the recognition of the systemic inflammatory response syndrome (SIRS) and the implementation of damage control surgery (DCS). Early acute temporary skeletal orthopaedic fixation is undertaken to stop bleeding from fractured cancellous bone. To minimise bone-bleeding the fractures should be realigned through the use of external fixation (EXFIX), skeletal traction, C-clamp, and percutaneous screws according to the fracture pattern (18, 38). In patients that had fractures amenable to minimally invasive internal fixation an early open reduction and internal fixation (ORIF) can be attempted, these include an iliosacral screw fixation or symphyseal plating, although choice of stabilisation technique is determined by the availability of resources and the training of the orthopaedic surgeon (44, 45).

Emergency trauma laparotomy is performed if there is a concern for intraabdominal bleeding (positive eFAST/DPA or continued haemodynamic instability). DCS to stop haemorrhage, eliminate contamination and stabilise the patient. Faecal diversion surgery may be required to prevent wound contamination (excluding any colon/rectal injury through stapling bowel proximal to injury) (16, 18, 23). An SPC can also be inserted during this initial operation, as definitive management of urogenital injuries is often delayed. An indwelling catheter (IDC) may also be required however this should only be attempted by an experienced practitioner (Urologist) if urethral injury is suspected and is usually done in conjunction with a retrograde cystoscopy and urethrogram in the definitive treatment phase (1). Advances in complex wound management with the use of vacuum-assisted closure (VAC) dressings (2, 46), and infection prevention (antibiotics, wound debridement, and irrigation). Angiography is also important to control arterial bleeding and if not immediately available then post DCS the patient may require further angiographic intervention. These measures have all contributed to the survivability of open pelvic fractures preventing late deaths due to sepsis. Interventional angiography is becoming more popular as a treatment modality and remains dependent on the availability and training of the interventional radiologists and the time to mobilise this service impact its use. Ideally the advent of the hybrid operating suite allows for simultaneous operative and angiography interventions, reducing the time to haemorrhage control and preventing further complications associated with TIC, allowing simultaneous diagnosis and treatment.

### Definitive Treatment Phase

Once the life-threatening haemorrhage has been arrested and the patient's physiology has been restored, definitive surgical treatments can be commenced. This is where co-ordination of sub-specialty individualised and needs based care becomes crucial to good patient outcomes, and this process should be adapted according to the patient's injuries and needs. These include; reconstructive procedures to provide soft tissue coverage including skin grafting and flaps, formal bowel diversion surgery (stoma for a colostomy) (15, 47–50), assessment of urethral injury (cystography and insertion of an IDC for urethral realignment, often definitive repair is delayed) (37), continued wound management with washouts and surgical debridement's of wounds and wound management using VAC dressings and instillation VAC therapy (VERAFLO) as required (2, 46, 51, 52).

Despite these advances, outcomes remain pessimistic as patients are left with life-altering consequences such as; chronic pain, physical disability, incontinence, sexual dysfunction, post-traumatic stress disorder (PTSD), and chronic infections (1, 3).

## Research Gaps

**Figure 3:**
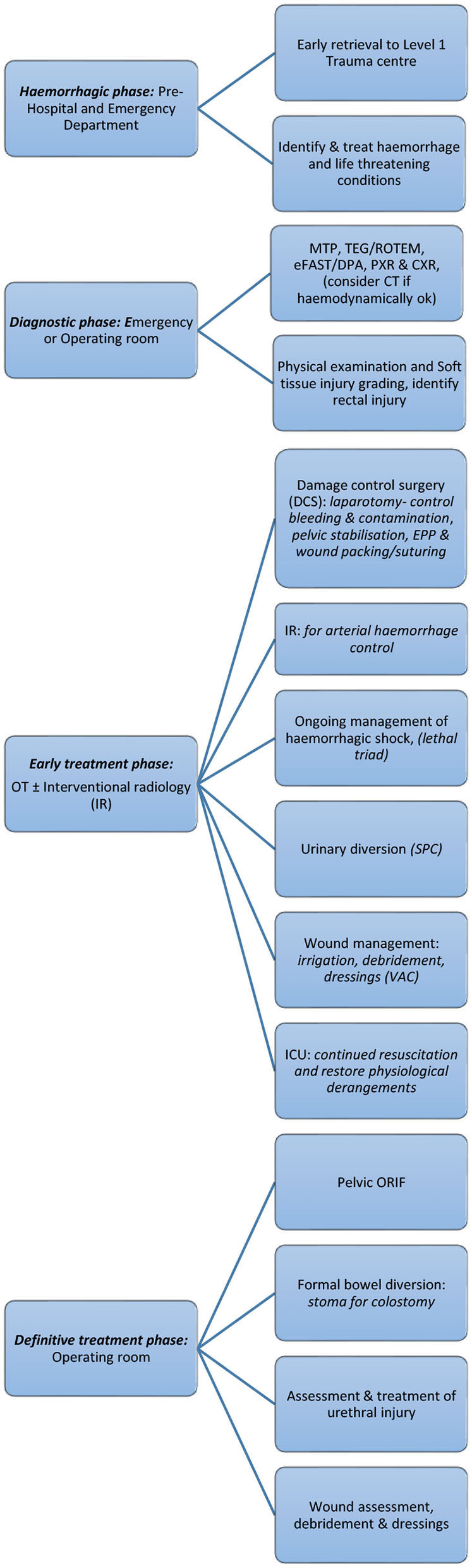
Open pelvic fracture management algorithm (1).

Less clear are specific management factors associated with morbidity and mortality in patients with open pelvic fractures and there is very little data available describing open pelvic fractures. Research to identify these factors has the potential to improve outcome for patients with this challenging clinical problem. The impact of advances in systems and technology to treat open pelvic fractures and the impact on morbidity and mortality warrants further exploration. A proposed management algorithm for open pelvic fractures based on existing literature is shown in Figure 3. Standard pelvic fracture and exsanguinating pelvic fracture algorithms do not sufficiently address the differences, priorities, and critical decision points associated with the acute management of open pelvic fractures.

To date, published studies on outcomes associated with open pelvic fractures have explored small heterogeneous samples in international settings with variously reported mortality rates. These studies report inconsistent approaches to each phase of open pelvic fractures management. Early and definitive treatments have contributed to improved survivability of open pelvic fractures although outcomes associated with the recovery phase are less optimistic. Chronic pain, physical disability, incontinence, sexual dysfunction, PTSD, and chronic infections are the unfortunate morbidity associated with open pelvic fractures. Scrutiny of factors linked to each phase of management is required to identify areas of variability in practice and benchmark best practice (1, 3).

## Future Developments

There is promise in the development of tissue engineering technology for application in the management of traumatic pelvic wounds to repair skin defects unable to be repaired using traditional techniques such as; autogenous flaps and bone, skin, and vessel grafts. Artificial skin, dermal substitutes, bioactive glasses, stem cell technology, and advanced wound dressings may bring hope to those suffering from chronic wounds and may prevent the formation of scar tissue during the healing process, promoting faster more effective wound coverage (24, 28, 46). This may also reduce morbidity from prolonged wound healing and infection causing sepsis.

The development of three-dimensional (3D) printing is also an area of interest as currently implants are widely used to repair bony defects of the pelvis and acetabulum especially in tumour surgery and revision hip surgery. Customisable bone replacements can be used to restore a bony defect to the correct anatomical position, size, and strength. However, the use of implants in open pelvic fractures carries significant risk of infection, due to contamination and tissue loss, and is not a good choice at present in this group of patients. Future developments, such as the use of antibiotic impregnated demineralised bone matrix may increase the application of implants in patients with open fractures (53). Research into the use of 3D printed scaffolds embedded with tissue growth factors to promote regeneration and tissue growth combines the tissue engineering technology with the 3D printing to produce faster healing that would be both functional and cosmetically acceptable (54, 55). However, there are currently many challenges with controlling the growth of the new tissue to obtain acceptable coverage of wounds without infection.

Wound management techniques also continue to develop and advances in machine technology, wound cleansing and infection prevention has also shown to promote wound healing leading to improved patient outcomes. This is an area of continued research and development that will provide new technology for wound management into the future.

Further developments can be expected from research into haemorrhage control in trauma including; TEG guided transfusion protocols, changes to haemostatic products such as blood substitutes and blood products for administration, drugs to combat TIC and advances in surgical techniques for early definitive fracture stabilisation.

## Author Contributions

RW was responsible for the literature review and formulation of the manuscript. JH was responsible for reviewing and revising the manuscript. All authors contributed to the article and approved the submitted version.

## Conflict of Interest

The authors declare that the research was conducted in the absence of any commercial or financial relationships that could be construed as a potential conflict of interest.
